# Development and psychometric testing of the Acceptability regarding Cognitive Rehabilitation Interventions Survey – Cancer Survivors (ACRIS-CS)

**DOI:** 10.1192/j.eurpsy.2024.439

**Published:** 2024-08-27

**Authors:** A. F. Oliveira, A. Bártolo, L. Loureiro, I. M. Santos, A. Torres

**Affiliations:** ^1^Department of Education and Psychology, Center for Health Technology and Services Research of the Health Research Network (CINTESIS@RISE), University of Aveiro, Aveiro; ^2^I2P - Portucalense Institute for Psychology, Portucalense University, Porto; ^3^Center for Global Studies, Open University, Lisboa; ^4^Department of Education and Psychology, University of Aveiro; ^5^Department of Education and Psychology, William James Center for Research (WJCR), University of Aveiro, Aveiro, Portugal

## Abstract

**Introduction:**

Cognitive rehabilitation interventions (CRIs) for cancer-related cognitive impairment (CRCI) have shown promising results. However, the acceptability of CRIs in the context of CRCI treatment has not yet been assessed among cancer survivors. Due to the absence of suitable instruments designed to assess the acceptability of CRIs in this population, we developed the Acceptability regarding Cognitive Rehabilitation Interventions Survey for Cancer Survivors (ACRIS-CS).

**Objectives:**

This study aimed to develop and test the psychometric properties of the newly created instrument, ACRIS-CS.

**Methods:**

The study was conducted in two stages: (1) the creation of scale items derived from a comprehensive literature review, considering the Theoretical Framework of Acceptability (TFA); and (2) the assessment of the scale’s psychometric properties with cancer survivors. At the end of stage 1, the questionnaire was revised by four clinicians and researchers with expertise in the field of CRCI, and the final item selection was determined by the authors, considering redundancy, item relevance, and face validity. The final scale comprised 11 items, answered on a 5-point Likert scale (ranging from “strongly disagree” to “strongly agree”). Higher scores indicated more positive perceptions related to the acceptability of CRIs. Data were collected online and analyzed using IBM SPSS Statistics (version 28.0). Construct validity (exploratory factor analysis, EFA) and reliability (internal consistency) analyses were performed.

**Results:**

In this study, 154 cancer survivors were included. The Kaiser-Meyer-Olkin (KMO) measure of 0.847 confirmed the adequacy of sampling (KMO>0.5), and Bartlett’s test of sphericity yielded statistical significance (Χ² (55) = 864.431, p < 0.001), validating the structure of the correlation matrix. The EFA results indicated the presence of three factors, each with eigenvalues exceeding the Kaiser criterion of 1. The scree plot confirmed the existence of three factors beyond the inflection point. All items demonstrated factor loadings higher than 0.40, indicating their relevance to the identified factors. This factor structure was conceptually justifiable. These factors were labeled as follows: 1) Affective attitude and effectiveness (6 items); 2) Perceived benefits and self-efficacy (3 items); and 3) Perceived burden (2 items). Collectively, these factors accounted for 68.7% of the total variance. The ACRIS-CS total scale and subscales demonstrated good internal consistency, with Cronbach’s alpha coefficients ranging from 0.727 to 0.848.

**Conclusions:**

The results of the EFA and internal consistency analysis were satisfactory. The ACRIS-CS appears to be a valid and reliable scale for assessing the acceptability of CRIs among cancer survivors.

**Disclosure of Interest:**

None Declared

